# DJ-1 Deficiency Protects against Sepsis-Induced Myocardial Depression

**DOI:** 10.3390/antiox12030561

**Published:** 2023-02-24

**Authors:** James N. Tsoporis, Hajera Amatullah, Sahil Gupta, Shehla Izhar, Amin M. Ektesabi, Chirag M. Vaswani, Jean-Francois Desjardins, Golam Kabir, Ana Paula Teixera Monteiro, Amir K. Varkouhi, Nikolaos Kavantzas, Vasileios Salpeas, Ioannis Rizos, John C. Marshall, Thomas G. Parker, Howard Leong-Poi, Claudia C. dos Santos

**Affiliations:** 1 The Keenan Research Centre of the Li Ka Shing Knowledge Institute of St. Michael’s Hospital, Unity Health Toronto, 30 Bond Street, Toronto, ON M5B 1W8, Canada; 2 Department of Physiology, Faculty of Medicine, University of Toronto, Toronto, ON M5S 1A8, Canada; 3 Institute of Medical Sciences, University of Toronto, Toronto, ON M5S 1A8, Canada; 4 1st Department of Pathology, School of Medicine, National and Kapodistrian University of Athens, 11528 Athens, Greece; 5 2nd Department of Cardiology, Attikon University Hospital, 12462 Athens, Greece

**Keywords:** DJ-1, sepsis, cardiomyocyte, inflammation, oxidative stress, autophagy

## Abstract

Oxidative stress is considered one of the early underlying contributors of sepsis-induced myocardial depression. DJ-1, also known as PARK7, has a well-established role as an antioxidant. We have previously shown, in a clinically relevant model of polymicrobial sepsis, DJ-1 deficiency improved survival and bacterial clearance by decreasing ROS production. In the present study, we investigated the role of DJ-1 in sepsis-induced myocardial depression. Here we compared wildtype (WT) with DJ-1 deficient mice at 24 and 48 h after cecal ligation and puncture (CLP). In WT mice, DJ-1 was increased in the myocardium post-CLP. DJ-1 deficient mice, despite enhanced inflammatory and oxidative responses, had an attenuated hypertrophic phenotype, less apoptosis, improved mitochondrial function, and autophagy, that was associated with preservation of myocardial function and improved survival compared to WT mice post-CLP. Collectively, these results identify DJ-1 as a regulator of myocardial function and as such, makes it an attractive therapeutic target in the treatment of early sepsis-induced myocardial depression.

## 1. Introduction

Sepsis is a complex disease process wherein the body’s response to a pathogen is profoundly dysregulated [1]. A recent WHO resolution recognizes sepsis as a Global Health priority. Current estimates are >30 million episodes and 6 million deaths per year. Currently, no specific treatment exists [2]. Patients receive antibiotics, source control, and supportive care [3,4]. Myocardial depression occurs in almost 40% of septic patients, is an important feature of multiorgan dysfunction syndrome (MODS), and is associated with increased morbidity, mortality, and poor long-term outcomes [5,6,7,8]. The importance of myocardial depression in sepsis has been recognized and associated with echocardiographic evidence of cardiac dysfunction and a low cardiac index [9,10]. Importantly, approximately, 60% of patients present a clinical picture of cardiovascular impairment with mortality ranging from 70–90%, in contrast with 20% mortality in septic patients without myocardial involvement [11]. Beyond ionotropic support, pharmacological approaches to alleviate sepsis-induced myocardial depression do not currently exist. Survivors of sepsis experience profound and potentially debilitating functional and cognitive impairment as well as accelerated heart disease [11,12]. It has been reported that many mechanisms participate in myocardial dysfunction induced by sepsis, including mitochondrial dysfunction, inflammation, oxidative stress, complement activation, apoptosis, and autophagy/mitophagy [8,13,14,15,16,17].

PARK7/DJ-1 (Parkinsonism associated deglycase) was initially identified as an oncoprotein [18]. The gene product contributes to familial early onset and sporadic forms of Parkinson Disease PD [19]. The many functions of DJ-1 include transcription regulation, protein stabilization, and anti-oxidative defense [20,21]. Knockout (KO) of DJ-1 increases susceptibility to oxidative stress [22,23]. Transaortic banding of DJ-1KO mice resulted in cardiac hypertrophy and heart failure [24]. In response to ischemia reperfusion injury, DJ-1KO mice displayed increased areas of infarction and worsened left ventricular function when compared to WT mice, confirming a protective role for DJ-1 in the heart [25,26]. In these animal models of cardiovascular disease, DJ-1 is required for normal cardiac function. However, in a model of polymicrobial sepsis (cecal ligation and puncture—CLP), DJ-1KO mice had improved bacterial clearance and survival [27]. Remarkably, DJ-1KO macrophages showed bactericidal activity and enhanced phagocytosis in vitro, and wild-type mice were rescued from CLP–induced mortality by the adoptive transfer of DJ-1KO bone marrow–derived mononuclear cells. In stimulated bone marrow-derived macrophages, DJ-1 inhibited the production of reactive oxygen species (ROS) by binding to the nicotinamide adenine dinucleotide phosphate (NADPH) complex component p47^phox^, disrupting the complex and facilitating the ubiquitination and degradation of Nox2 (gp91^phox^). In THP-1 (human monocytic cell line) and polymorphonuclear cells from patients with sepsis, knock down of DJ-1 improved bacterial killing and respiratory burst. Interestingly, in septic patients, DJ-1 plasma levels were increased. Rubicon (Run domain Beclin-1-interacting and cysteine-rich domain-containing protein), a component of the Class III PI3K complex, functions as a negative regulator of autophagy [28]. We demonstrated that during bacterial infection, DJ-1 deficiency in bone marrow-derived macrophages elicits a Rubicon-dependent increase in LC3-associated phagocytosis, a non-canonical autophagy pathway used for xenophagy [29]. Our data suggesting a protective effect of DJ-1 deficiency in sepsis is in direct contrast to studies suggesting that loss of DJ-1 exacerbates cardiac pathologies and may depend on the experimental model and tissues investigated. In the present study, we address the role of DJ-1 in sepsis-induced myocardial dysfunction.

## 2. Materials and Methods

### 2.1. Bioinformatics Analysis

We have previously published network analysis of transcriptional responses induced by mesenchymal stem cell treatment of experimental sepsis (GEO ID: 40180) [30]. In brief, in C57Bl/6J mice, we induced sepsis by cecal ligation and puncture, followed by an i.v. injection of either MSCs or saline 6 h later. Total RNA harvested from hearts 28 h after CLP was hybridized to mouse expression bead arrays. Common transcriptional responses were analyzed using a network knowledge-based approach. Using the Robust Multi-array Average (RMA) method, the microarray was normalized and differentially expressed (DE) genes (*p* value < 0.05 & FDR < 0.05) were recognized using limma package from Bioconductor. Enrichr ( http://amp.pharm.mssm.edu/Enrichr/ (accessed on 1 March 2021)) was used to calculate pathway enrichment within the DE genes. The gplot package was used to generate heatmaps.

### 2.2. Human Samples

Archival blocks of formalin-fixed, paraffin-embedded heart tissue from 10 patients who did from acute respiratory distress syndrome (6 male and 4 female; mean age 55 ± 9 and 60 ± 16 years, respectively) and 5 control patients who died from cancer (lung negative for malignancy) (3 male and 2 female; mean age 62 ± 3 and 48 years, respectively) were obtained through a collaboration (research ethic board protocol # 10/2021) with the 1st Department of Pathology, School of Medicine, National and Kapodistrian, University of Athens, Greece.

### 2.3. Animals

All protocols were approved by the Animal Care Committee (ACC#476, dated 25 June 2013) at Unity Health-St. Michael’s Hospital (Toronto, ON, Canada). Wildtype (WT, C57Bl/6J, Jackson Laboratories), and DJ-1 deficient mice (targeted deletion of DJ-1, DJ-1KO [28]) on a C57Bl/6J background (20 backcrosses) were housed in a specific pathogen free facility with standard 12-h light/dark cycle with *ad libitum* access to Teklan Global 18% Protein Rodent Diet and RO water. Mice were acclimatized to the animal facilities for at least a week before beginning experiments. Mice were treated humanely in accordance with the guidelines outlined in the Canadian Council of Animal Care. DJ-1 deficient mice were generated previously and obtained from Dr. Tak W. Mak’s laboratory. Details of the creation of the transgenic mouse have been previously characterized [31]. Briefly, exons 3–7 were excised, and the first coding exon was modified to contain a premature codon. Absence of DJ-1 mRNA and protein was also documented in our studies below.

### 2.4. CLP Model of Sepsis

We have established a model of hemodynamically stable sepsis in mice [27]. The mice were randomized into treatment groups and all assessors blinded to treatment group assignment. We used the CLP model to reproduce the cardiovascular pathophysiology of sepsis and assess the expression of the DJ-1 [27]. In brief, the cecum was exteriorized and ligated 1 cm from the apex with 3–0 silk suture, and with an 18-gauge needle penetrated through-and-through and incision was closed. The cecum was exteriorized and manipulated but not ligated or punctured in control sham surgeries. Subcutaneous fluid resuscitation with 50 mL/kg saline and 0.2 mg/kg Buprenorphine (Buprenex) was administered twice daily to all animals. Animals were placed in a temperature-controlled incubator and monitored every 6 h for the first 48 h; monitoring was de-escalated to every 8 h until the end of the experiments. Welfare-related assessment scores included reflexes, motor activity, and overall gross appearance.

### 2.5. Echo and Hemodynamic Monitoring

Forty-eight hours after surgery, mice were anesthetized with 3–5% isoflurane and echocardiography imaging, with a 12-mHz probe (Hewlett Packard Sonos 5500, Philips, Bothell), was performed, as described previously [32]. Ejection fraction (EF) (%) = ((LVEDD-LVESD)3/LVEDD3) × 100 and fractional shortening (FS) (%) = ((LVEDD-LVESD)/LVEDD) × 100 were calculated where LVEDD and LVESD are left ventricular end diastolic and systolic diameter(s), respectively, as previously described [32]. Three consecutive cardiac cycles were averaged for all analyses. For in situ hemodynamics, a 1.4F pressure–volume (PV) catheter (SPR-838; Millar Instruments, Inc., Houston, TX) was inserted into the right carotid of the animal and advanced into the left ventricle to generate PV loops. All pressure–volume (PV) loops were obtained with the ventilator turned off for 5–10 s and the animal apneic [32].

### 2.6. Tissue Collection

Mice were sacrificed following 24 and 48 h post-CLP/sham and tissues harvested for analysis. Whole blood was collected by cardiac puncture, centrifuged, and serum separated for biochemical and mediator analysis. The heart was formalin-fixed for histology or snapped-frozen and stored at −80 °C for protein and RNA analyses.

### 2.7. Cell Culture

Cardiac myocytes were dissociated from 1- to 2-day-old wildtype (WT, DJ-1KO) C57Bl/6J (Jackson Laboratories) and DJ-1 knock out mice hearts (DJ-1KO) by trypsinization, with a yield of approximately 5 × 10^6^ per heart, as previously described [33]. The cells were changed to serum-free medium before initiation of each experiment. Cell cultures were treated with 1 µg/mL of lipopolysaccharide (LPS) (*E. coli* LPS 026:B6 and 055:B5, Sigma-Aldrich, St. Louis, MO, USA) for 24 h [27]. Total protein in cell lysates was estimated by Bradford Assay. Transfection of cardiac myocytes with a DJ-1 expression plasmid (SwitchGear Genomics, Menlo Park, CA, USA) and/or Rous sarcoma virus–luciferase (as an internal control for transfection efficiency) was performed with FuGENE 6 reagent, according to the manufacturer’s instructions (Roche, Indianapolis, IN, USA), using 1 μg of each plasmid. Caspase-3 activity was measured from cell lysates using EnzChek Caspase-3 assay kit (Invitrogen, Carlsbad, CA, USA).

### 2.8. Real-Time Quantitative RT-PCR

RNA was extracted from tissues and cell cultures as previously described [26,29]. Gene specific sequences of oligonucleotide primers were purchased from Qiagen (Alameda, CA, USA). Real time quantitative RT-PCR was performed according to the manufacturer’s protocol (Qiagen, Alameda, CA, USA). Briefly, a 25 μL reaction volume containing 10.5 μL of H_2_O, 12.5 μL of RT^2^ SYBR Green qPCR Master Mix, 1 μL of gene-specific 10 μM PCR primer pair stock of gene of interest (proprietary sequences) [mouse-18S, DJ-1, Myh6 (α myosin heavy chain), Myh7 (β myosin heavy chain), HMOX-1 (heme oxidase 1), Nrf2 (nuclear factor erythroid 2), PGC1-α (peroxisome proliferator-activated receptor gamma coactivator 1-alpha), NADPH, GPx1 (glutathione peroxidase 1), GSTs (glutathione s transferase), TNF-α (tumor necrosis factor 1 alpha), IL-6 (interleukin 6), IL-1β, ANF (atrial natriuretic factor), S100A1, S100A8, S100A9, BAX, BCL2, XAIP, NAIP, and human (proprietary sequences)—18S, DJ-1, IL-6, IL-1β, and Beclin1 1 μL of RT^2^ First-Strand cDNA (template) underwent a two-step cycling program; 40 cycles, 10 min at 95 °C, 15 s at 95 °C, and 1 min at 60 °C (Qiagen, Alameda, CA, USA). In separate experiments, the threshold cycle (C_t_) value for the housekeeping gene 18S and gene of interest per sample was determined. Only C_t_ values less than 35 were used. The difference between the C_t_ values (ΔCt) for each gene of interest and 18S was calculated and the fold-change in gene expression was calculated using the equation 2^(−ΔΔCt)^ [26,29]. The mean-fold change for each gene in the experimental group is shown relative to the gene in the control/sham group that is set to 1.

### 2.9. Histopathology Assessment

Hearts were fixed and processed into paraffin-embedded blocks and sectioned as described [26,29]. Slides were stained with hematoxylin and eosin for histopathological assessment. For immunohistochemistry, tissue sections were deparaffinized and hydrated with ethanol washes. For antigen retrieval, slides were incubated with primary antibody (DJ-1, #5933-Cell Signaling, 1:1000) overnight at 4 °C. Slides were developed with 3,3’Diaminobenzidine (DAB )substrate and counterstained with hematoxylin and viewed with an Olympus upright BX50 microscope.

### 2.10. Transmission Electron Microscopy (TEM)

Samples for TEM were fixed, sectioned and stained as previously described [29]. Images were viewed with a FEI Tecnai 20 TEM. Mitochondrial density was calculated as a percentage of myocardial cell cytoplasm.

### 2.11. TUNEL Staining

Terminal dUTP nick end-labeling (TUNEL) nuclei were visualized at X400, as previously described [32,33]. In brief, apoptotic cells in left ventricular (LV) sections were detected by labelling of nuclear DNA strand breaks with the DeadEnd Fluometric TUNEL System (Promega, Madison, WI, USA). The apoptotic index was calculated as TUNEL + nuclei × 100/DAPI + nuclei.

### 2.12. Western Blot

Western blotting was performed as described previously [27]. Tissue and cell lysates were probed with the following antibodies at a concentration of 1:1000: mouse DJ-1 (sc#55572), mouse β actin (sc#69879), mouse PGC-1α (sc#517380), (Santa Cruz, CA, USA), rabbit HMOX-1 (#86806, Cell Signaling, Danvers MA), rabbit eNOS (#32027, Cell Signaling), rabbit iNOS (#ab178945, Abcam, Cambridge, MA, USA), rabbit p62 (#H0023636-P01, Abnova, Walnut, CA, USA), rabbit LC3 (#D3U4C, Cell Signaling), and rabbit Rubicon (#8465, Cell Signaling) for an hour at room temperature or overnight at 4 °C, and then incubated with horseradish peroxidase conjugated secondary antibodies for an hour, using Bio-Rad Gel Doc 2000. Densitometry was performed using Gel-Quant-Net software. Plasma DJ-1 was measured by enzyme-linked immunosorbent assay ( ELISA )(#DY3995, R&D Systems, Minneapolis, MN, USA) according to the manufacturer’s instructions. Assay range:62.5–4000 pg/mL.

### 2.13. Serum Biochemistry Markers

VetScan Comprehensive Diagnostic Profile (VetScan Test Panels, University Health Network, Toronto, ON, Canada) was used to measures serum levels of albumin, alkaline phosphatase (ALP), alanine transaminase (ALT), blood urea nitrogen (BUN), total bilirubin, amylase, and glucose. Serum cardiac troponin I (cTnI) levels were measured using commercially available High Sensitivity Mouse Cardiac Troponin-I Elisa Kit (Cat. No. 2010-1-HS by Life Diagnostics, Inc., West Chester, PA, USA).

### 2.14. Levels of Inflammatory Mediators

The levels of inflammatory marker in myocardial were measured using a custom murine specific Procarta Cytometric Bead Array (Affymetrix Panomics, Santa Clara, CA, USA), according to the instructions of the manufacturer.

### 2.15. Tissue ROS Measurements

For reactive oxygen species (ROS) measurement 24- and 48-h post-CLP study, the hearts were homogenized in phosphate-buffered saline (PBS), and dichlorofluprescein (DCF) fluorescence was measured using the OxiSelect ROS/RNS assay kit (Cat. No. STA-347, Cell BioLabs Inc., Burlington, ON, Canada), according to manufacturer’s instructions. Values were normalized to protein input.

### 2.16. Statistical Analyses

Mice were randomized to treatment groups and investigators were blinded to genotype and group assignment. Survival curves were generated using Log Rank (Mantel-Cox) tests. Data are presented as mean + standard error of mean (SEM). The Mann–Whitney was used to determine differences between 2 groups, and for multiple groups, ANOVA followed by Bonferroni post-hoc test was carried out. GraphPad PRISM 6.0 was used for analysis.

## 3. Results

### 3.1. Bioinformatic Analysis

**Figure 1:**
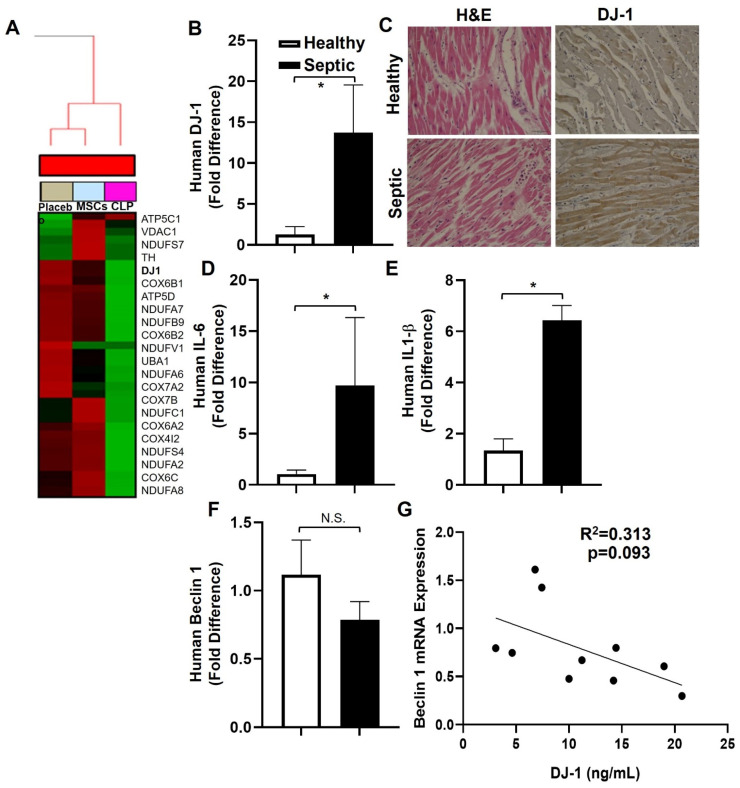
Identification of DJ-1/PARK7 through Bioinformatic Analyses and Expression in Human Septic Patients. (**A**) Gene expression profile of Kegg Parkinson’s disease gene-set in the heart after treatment with MSCs. The data used was from LIMMA analysis (N = 45,281 genes) comparing CLP + Placebo vs. CLP + MSCs and identified through Gene Set Enrichment Analysis (GSEA). (**B**) Bars are DJ-1/18S mRNA expression + SEM in in archival myocardial samples from patients deceased from severe sepsis with myocardial dysfunction and myocardial (non-malignant) tissue from cancer patients serving as control. (**C**) Representative photomicrographs (×400) sections of formalin-fixed paraffin-embedded myocardia tissue from a human subject (male, 58 years old), who died from cancer, serving as control and a human subject (male, 44 years old) deceased from severe sepsis with myocardial dysfunction showing H&E staining and cytosolic expression of DJ-1. Bar = 20 μ. (**D**–**F**) Bars are mean + SEM of gene/18S mRNA expression in archival myocardial samples of patients who died from severe sepsis with myocardial dysfunction and myocardial (non-malignant) tissue from cancer patients serving as control. *n* = 5–10, N.S. – not significant, * *p* < 0.05. (**G**) Correlation of septic DJ-1 vs. beclin 1mRNA levels. *n* = 10.

Gene set enrichment analysis (GSEA) identified a total of 4751 genes significantly changed in the heart between CLP− vs.CLP + MSCs-treated mice (adjusted *p* ≤ 0.05), using LIMMA package from Bioconductor. Using Enrichr, we calculated pathway enrichment within the differentially expressed genes and identified Parkinson disease (PD) associated genes as the top enriched pathway. Within the Parkinson disease pathway, the top 25 gene signatures showed dysregulation of gene expression compared to the placebo (Figure 1A). CLP induced the upregulation of genes involved in inflammatory pathways (COX gene family), NADH oxoreductase-associated genes (NUDF family) and antioxidants (PARK7/DJ-1), and CLP + MSCs downregulated the PD gene signature (Figure 1A).

### 3.2. D-1-, IL-6, and IL-1β Are Increased in Human Septic Hearts

We extracted RNA from formalin fixed paraffin embedded heart autopsy sections from deceased patients who died from sepsis or from cancer (lung negative for malignancy), proven by biopsy, that served as controls. Semi-quantitative RT- PCR showed that the mRNA expression of DJ-1, IL-6, and IL-1β were increased in septic hearts compared to controls (Figure 1B,D,E). Immunohistochemistry showed increased DJ-1 expression in septic myocardial tissue compared to the control (Figure 1C). Interestingly, there was a trend for decreased mRNA expression of the autophagy effector beclin1 in septic hearts (Figure 1F), and a trend of a negative correlation between DJ-1 vs. beclin1 mRNA levels (Figure 1G).

### 3.3. DJ-1 Deficiency Improves Survival Post-CLP

**Figure 2:**
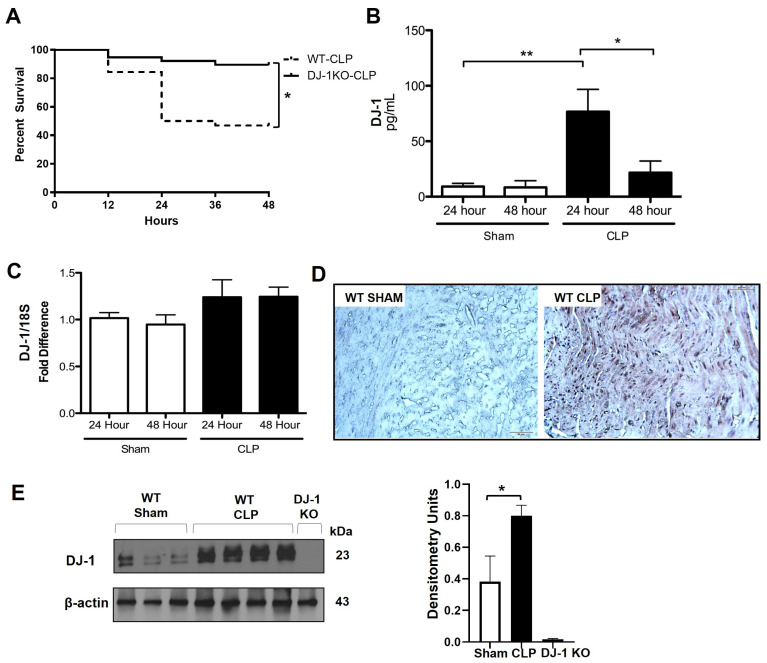
DJ-1 is increased in myocardium post-CLP. (**A**) Percent survival at 48 h post-CLP in WT and DJ-1KO (*n* = 11). (**B**) Bars are mean + SEM of serum DJ-1 (ng/mL) of WT and DJ-1KO mice at 24 and 48 h post-CLP or sham operation. *n* = 11. (**C**) Bars are mean + SEM of DJ-1/18S mRNA expression in myocardium of WT and DJ-1KO mice at 24 and 48 h post-CLP or sham operation. *n* = 11. (**D**) Representative histology photomicrographs (×400) sections of formalin-fixed paraffin-embedded myocardial tissue from WT and DJ-1KO at 48 h post-CLP. Bar = 20μ. (**E**) Representative Western blot showing DJ-1 protein in myocardium from WT and DJ-1KO at 48 h post-CLP or sham operation. Densitometry units are shown. * *p* < 0.05; ** *p* < 0.01.

DJ-1 deficient mice compared to WT showed improved survival at 48 h and at 7-day post-CLP in fluid resuscitated, antibiotic treated mice, as previously described (Figure 2A) [26]. While WT mice became lethargic, stopped grooming, and showed moderate distress after CLP, DJ-1−/− mice showed no, or only mild distress. Body weight and temperature were not different between genotypes (data not shown). The poor survival of septic WT mice was associated with increased plasma DJ-1 protein at 24 and 48 post CLP, as confirmed by immunohistochemistry (Figure 2B). Next, we determined the expression of DJ-1 in the hearts of WT and DJ-1 deficient mice post CLP. Although WT animals did not show an increase in DJ-1 mRNA expression at 24 and 48 h following CLP surgery, DJ-1 protein was increased in WT mice 48 h post CLP, as confirmed by immunohistochemistry (Figure 2C,E).

### 3.4. DJ-1 Deletion Exacerbates Inflammation Post-CLP

**Figure 3:**
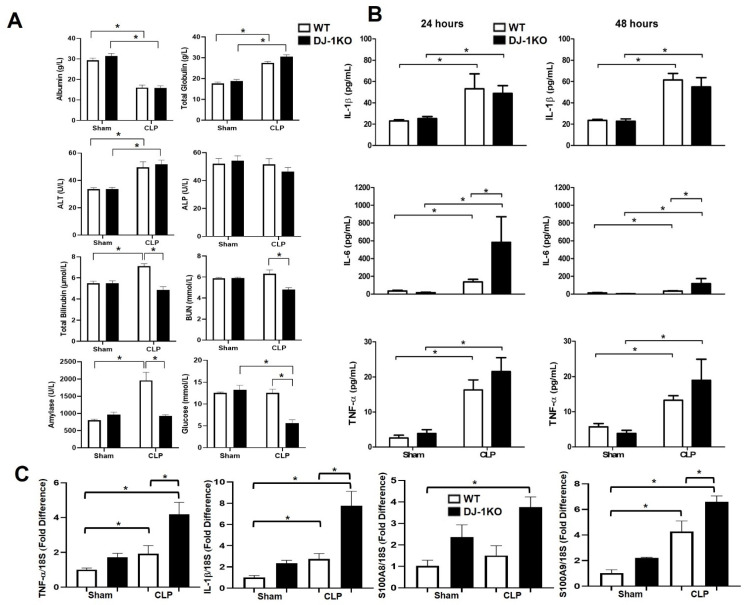
DJ-1 deficiency exacerbates inflammation post-CLP. (**A**) Bars are mean + SEM of VetScan-derived chemistry profiles of albumin, total globulin, alamine aminotransferase (ALT), alkaline phosphatase (ALP), total bilirubin, blood urea nitrogen (BUN), amylase, and glucose in serum of WT and DJ-1KO mice at 48 h post CLP or sham operation. *n* = 9–11/group. (**B**) Bars are mean + SEM of IL-1β, IL-6, or TNF-α in serum of WT and DJ-1KO mice at 48 h post CLP or sham operation. *n* = 9–11/group. (**C**) Bars are mean + SEM of TNF-α, IL-1β, S100A8, or S100A9 mRNA expression in myocardium of WT and DJ-1KO mice at 48 h post CLP or sham operation. *n* = 8–11/group. * *p* < 0.05.

Total globulin and alanine amino transferase (ALT) were increased whereas albumin was decreased equally in both genotypes. Total bilirubin, blood urea nitrogen (BUN), amylase, and glucose levels were lower in DJ-1KO compared to WT post-CLP mice (Figure 3A). The inflammatory cytokines TNF-α, IL-6, and IL-1β were increased in the serum of WT and DJ-1KO mice at 24 and 48 h post-CLP (Figure 3B). Moreover, IL-6 was increased to a greater extent in DJ-1KO compared to WT post-CLP at both time points (Figure 3B). In heart tissue, TNF-α and IL-1β mRNA levels were increased 48 h post-CLP, albeit to a greater extent in DJ-1KO (Figure 3C). Additionally, the expression levels of inflammatory S100 family members S100A8 and S100A9, also referred to as damage-associated molecular patterns (DAMPs), that signal cellular damage to resident cells via molecular pattern recognition receptors, were also increased 48 h post-CLP in the hearts of WT and DJ1KO (Figure 3C). These results confirm previous findings of DJ-1 as a regulator of inflammation [27]. More importantly, they delineate levels of inflammation with cardiac dysfunction.

### 3.5. DJ-1 Deficiency Protects against CLP-Induced Myocardial Depression

**Figure 4:**
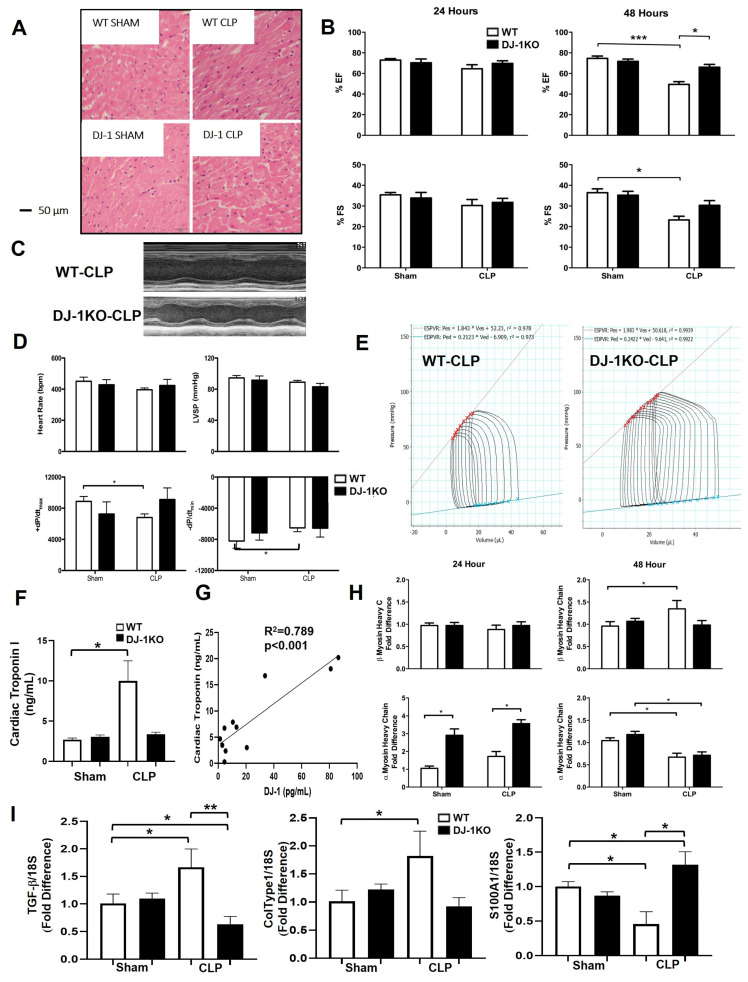
DJ-1KO mice are protected from sepsis-induced myocardial depression. (**A**) Representative immunohistochemical images of myocardium from WT and DJ-1KO mice at 48 h post-CLP or sham operation. (**B**) Representative m mode echocardiogram from WT and DJ-1KO mice at 48 h post-CLP. (**C**) Bars are mean + SEM of echo-derived ejection fraction (EF) or fractional shortening (FS) in WT and DJ-1KO mice at 24 and 48 h post CLP or sham operation. *n* = 9–11/group. (**D**) Bars are mean + SEM of heart rate (HR), LV systolic pressure, +dp/dtmax or -dp/dtmax of WT, and DJ-1-/- mice at 24 and 48 h post-CLP or sham operation. *n* = 9–11/group. (**E**) Representative PV loops from WT and DJ-1KO mice at 48 h post-CLP showing end systolic (ESPVR) and end diastolic (EDPVR) pressure volume relationships. (**F**) Bars are mean + SEM of plasma cardiac troponin (ng/mL) of WT and DJ-1KO mice at 24 h post-CLP or sham operation. *n* = 9–11/group. (**G**) A positive correlation between septic plasma DJ-1 (pg/mL) vs. cardiac troponin (ng/mL) at 24 h post-CLP. *n* = 11/group. (**H**) Bars are mean + SEM of β- or α-myosin heavy chain/18S mRNA expression in myocardium of WT and DJ-1KO mice at 24 and 48 h post-CLP or sham operation. *n* = 9–11/group. (**I)** Bars are mean + SEM of TGF-β, collagen (col) type I, or S100A1 mRNA expression in myocardium of WT and DJ-1KO mice at 48 h post-CLP or sham-operation. *n* = 9–11/group. * *p* < 0.05, ** *p* < 0.01, *** *p* < 0.001.

Next, we investigated cardiac structural and functional parameters by echocardiography and hemodynamic assessment at 24 and 48 h post CLP in WT and DJ-1. Histological analysis showed mild to moderate myofibrillary damage in the myocardium of WT but not DJ-1KO mice (Figure 4A). Alterations in myocardial function were not detected at 24 h post CLP in both WT and DJ-1KO (Figure 4C). At 48 h CLP, WT mice developed sepsis-induced myocardial depression as assessed by decreases in left ventricular ejection fraction (EF) and fractional shortening (FS) derived from M-mode echocardiography tracings (Figure 4B, C). In contrast, DJ-1KO mice did not develop myocardial depression post-CLP (Figure 4C). Hemodynamic assessment at 48 h post-CLP, showed no changes in heart rate and LV systolic pressure in both WT and DJ-1KO mice (Figure 4D). However, contractility (+dp/dt and −dp/dt) was reduced only in WT mice 48 h post-CLP (Figure 4D). When compared with WT, end-systolic pressure-volume relation (ESPVR) was increased in DJ-1KO post-CLP, indicative of improved cardiac contractility (Figure 4E). Plasma cardiac troponin levels only increased in the WT group 48 h post-CLP (Figure 4F). Interestingly, there was a positive correlation between septic plasma DJ-1 vs. cardiac troponin. These findings suggest that DJ-1 deficiency offers protection against CLP-induced myocardial depression. An examination of hypertrophic markers showed that the fetal isoform of β myosin heavy chain (MHC) associated with progressive myocardial dysfunction was induced only in the myocardium of WT mice at 48 h post CLP (Figure 4E). The absence of DJ-1 increased the expression of the adult isoform αMHC at 24 h (both in sham and CLP mice and waning off by 48 h (Figure 4E). The expression of markers of fibrosis, TGFβ, and collagen type 1 was also increased only in the myocardium of WT at 48 h post-CLP (Figure 3G). The S100 family member S100A1 is a regulator of sarcoplasmic reticulum Ca^2+^ handling as well as myofilament and mitochondrial function, thus enhancing the heart’s lusitropic and inotropic states [34]. In the myocardium of WT mice, CLP decreased S100A1 expression. Whereas in DJ-1KO, CLP increased the expression of myocardial S100A1 (Figure 4G). These results suggest that the abrogation of DJ-1 post-CLP preserves cardiac function.

### 3.6. DJ-1 Deficiency Suppresses Nitrative Stress Post-CLP

**Figure 5:**
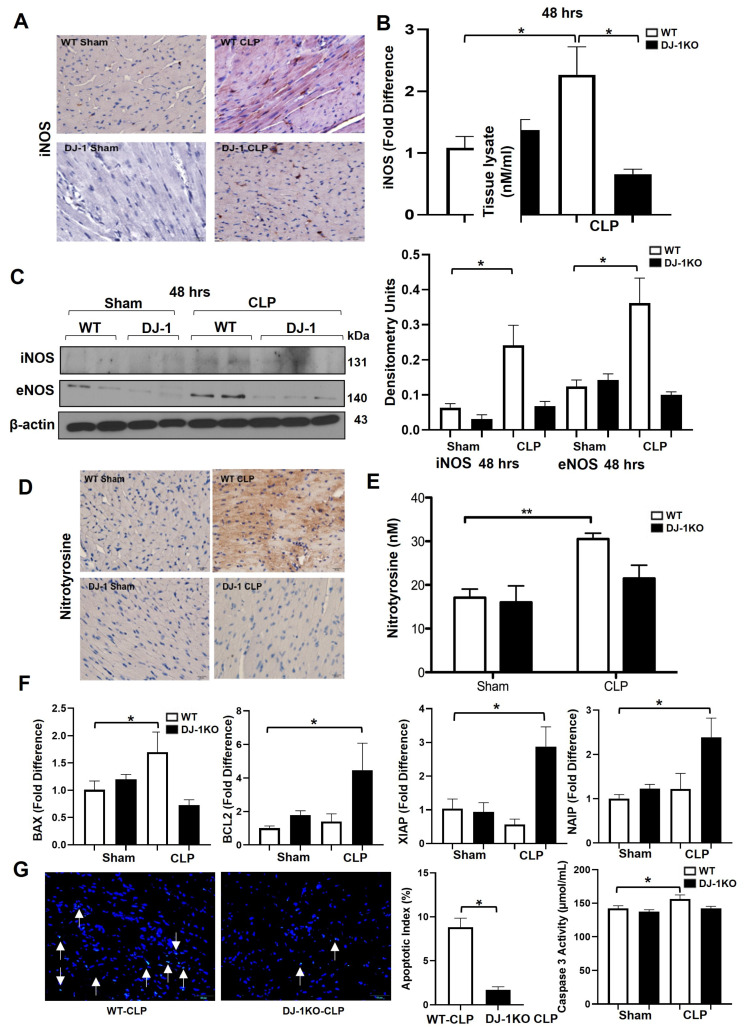
DJ-1 deficiency attenuates myocardial nytrosylation. (**A**) Representative immunostaining of iNOS in myocardium of WT and DJ-1KO mice at 48 h post CLP or sham operation. Bar = 20 m. (**B**) Bars are mean + SEM of iNOS- or eNOS/GAPDH mRNA expression in the myocardium of WT and DJ1KOmice at 48 h post-CLP or sham-operation. *n* = 9–11/group. (**C**) Representative Western blot of iNOS and eNOS in myocardium of WT and DJ-1KO mice at 48 h post-CLP or sham-operation. Densitometry units are shown. *n* = 3/group. (**D**) Representative immunohistochemical analysis of nitrotyrosine expression in myocardium of WT and DJ-1 mice at 48 h post-CLP or sham operation. Bar = 20 m. (**E**) Bars are mean + SEM of nitrotyrosine (nM) in myocardium of WT and DJ-1KO mice at 48 h post-CLP or sham operation. *n* = 8/group. (**F**) Bars are mean + SEM of BAX, BCL2, XIAP, or NAIP mRNA expression/18S in myocardium of WT and DJ-1KO mice at 24 and 48 h post-CLP or sham operation. *n* = 9–11/group. (**G**) Representative myocardial sections showing TUNEL positive nuclei of WT and DJ-1KO mice 48 h post-CLP. Bars are mean + SEM of Apoptotic index (%) and caspase 3 activity in the myocardium of WT and DJ-1KO mice 48 h post-CLP. * *p* < 0.05,.** *p* < 0.01.

To investigate the molecular mechanisms underlying the differences in cardiac outcomes, we next investigated the expression of inducible NOS (iNOS), which has been implicated in sepsis-induced myocardial depression [8]. Immunohistochemistry analyses of iNOS protein at 48 h confirmed attenuated expression of iNOS in DJ-1KO hearts compared to WT hearts, following CLP surgery (Figure 5A). Notably, iNOS expression in the DJ-1KO hearts appears to only be stained in infiltrating cells but not in parenchymal cells. Expression of iNOS mRNA and protein was increased in the myocardium of WT but not DJ-1KO mice 48 h post-CLP (Figure 5B,C). eNOS protein was similarly lower in DJ-1KO mice compared with WT mice at 48 h post-CLP (Figure 5C). Nitrotyrosine is a product of tyrosine nitration and an indicator of inflammation, nitric acid production, and cell damage. Immunohistochemistry analyses of nitrotyrosine protein at 48 h similarly revealed increased expression in WT hearts but attenuated expression in DJ-1KO hearts (Figure 5D). Nitrotyrosine levels in heart tissue lysates, as determined by ELISA, showed the same trend (Figure 5E). Next, we assessed markers of apoptosis. Proapoptotic BAX was upregulated at 48 h post-CLP in WT mice (Figure 5F). Members of the anti-apoptotic BLC2 family (BCL2, XIAP and NAIP) were selectively upregulated in the hearts of DJ-1KO 48 h post-CLP (Figure 5F). Moreover, there was an increase in myocardial TUNEL positive nuclei and caspase 3 activity in WT compared to DJ-1KO mice 48 h post-CLP. *n* = 9–11. * *p* < 0.05.

### 3.7. DJ-1 Deficiency Increases Antioxidant Pathways Post-CLP

**Figure 6:**
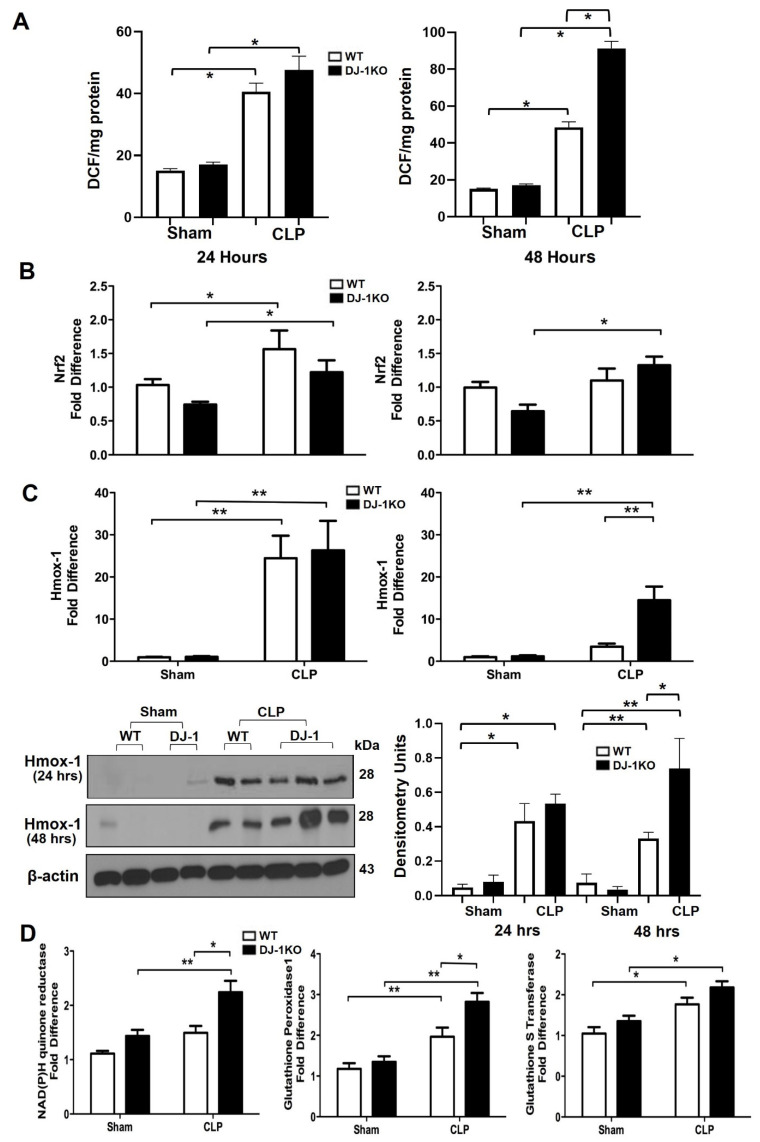
DJ-1 deficiency regulates myocardial oxidative stress post-CLP. (**A**) Bars are mean + SEM of myocardial ROS (DCF/mg myocardial protein) of WT and DJ-1KO mice at 24 and 48 h post-CLP or sham operation. *n* = 9–11/group. (**B**) Bars are mean + SEM of Nrf2 or HMOX-1 mRNA expression/18S myocardium of WT and DJ-1KO mice at 24 and 48 h post-CLP or sham operation. *n* = 9–11/group. (**C**) Representative western blot showing expression of Nrf2 and HMOX-1 protein in myocardium of WT and DJ-1KO mice at 24 and 48 h post-CLP or sham operation. Densitometry units are shown. *n* = 3/group. (**D**) Bars are mean + SEM of NADPH quinone oxidoreductase 1, glutathione peroxidase 1, or glutathione S transferase mRNA expression/18S in myocardium of WT and DJ-1KO mice at 24 and 48 h post-CLP or sham operation. *n* = 9–11/group. * *p* < 0.05, ** *p* < 0.01.

Next, we evaluated septic-induced myocardial ROS production and oxidative injury. As expected, increased myocardial ROS production was observed at 24 and 48 h post CLP in WT mice as previously noted (Figure 6A) [27]. Interestingly, DJ-1KO mice had enhanced ROS production at 48 h post CLP (Figure 6A). In non-canonical activation of Nrf2-dependent genes in DJ-1KO hearts following CLP surgery, Nrf2 is a master transcriptional regulator of the antioxidant response [35,36]. We have previously shown that DJ-1 can regulate Nrf2 activity via modulating its stability [27]. We assessed mRNA expression of Nrf2 and observed attenuated Nrf2 expression in DJ-1KO hearts, following CLP, compared with WT hearts at 24 h (Figure 6B). However, at 48 h, DJ-1KO mice had enhanced Nrf2 expression compared with WT hearts (Figure 6B). The mRNA expression of heme oxygenase-1 (HMOX-1), a key Nrf2-dependent gene, showed increased levels at 24 h for both WT and DJ-1KO hearts following CLP surgery (Figure 6C). At 48 h, DJ-1KO hearts continue to have enhanced HMOX-1 mRNA expression as levels declined in WT hearts (Figure 6C). Heme oxygenase protein expression showed a similar trend with enhanced levels at 48 h in DJ-1KO hearts (Figure 6C). We investigated the mRNA expression of other Nrf2- dependent genes, NAD(P)H quinone oxidoreductase 1 (NQO-1), glutathione peroxidase 1 (GPX-1), and glutathione s transferase (GSTs) at 48 h following CLP surgery. NQO1, GPX-1, and GSTs mRNA levels were enhanced in DJ-1KO hearts compared with WT following CLP-induced sepsis (Figure 6C). These findings suggest that Nrf2 dependent pathway is non-canonically activated in DJ-1KO hearts at a later point, independent of DJ-1′s positive regulation of Nrf2 expression.

### 3.8. DJ-1 Deficiency Exacerbates Mitochondrial Biogenesis and Attenuates Apoptosis Post-CLP

**Figure 7:**
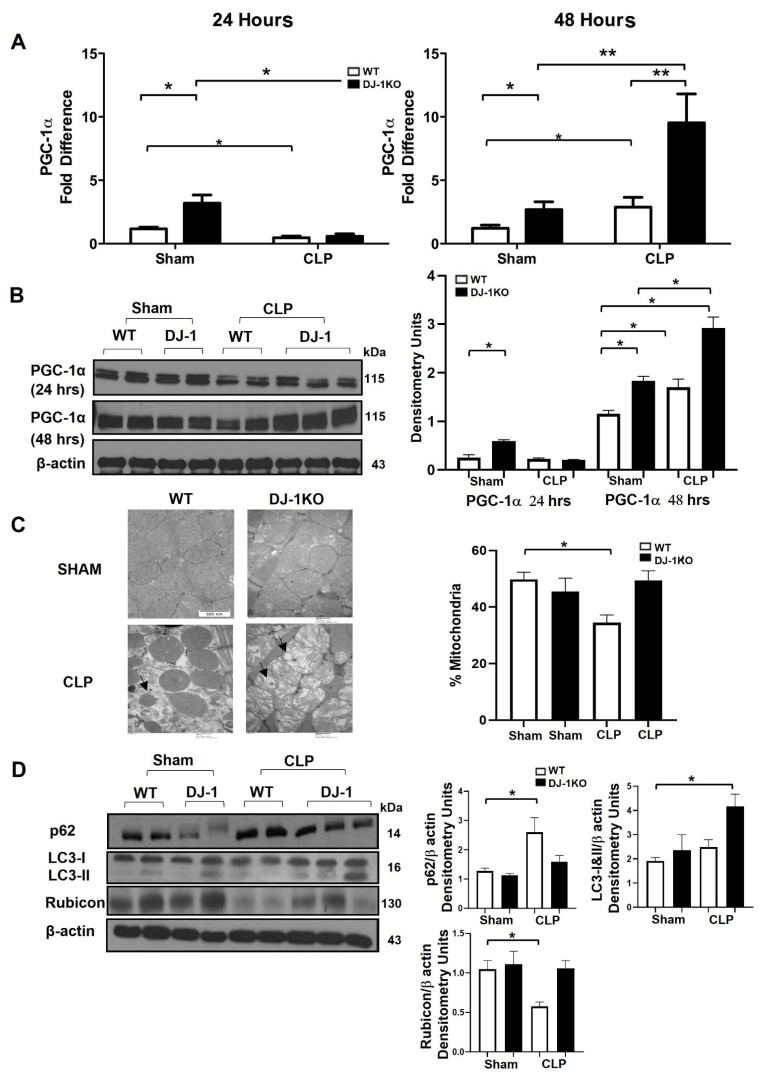
DJ-1 deficiency increases autophagy and decreases apoptosis. (**A**) Bars are mean + SEM of PGC-1a mRNA expression/18S in myocardium of WT and DJ-1KO mice at 24 and 48 h post-CLP or sham operation. *n* = 9–11/group. (**B**) Representative Western blots of PGC-1a protein expression in the myocardium of WT and DJ-1KO mice at 24 and 48 h post-CLP or sham operation. Densitometry units are shown. *n* = 3/group. (**C**) Representative transmission electron microscopy micrographs depicting double stranded autophagosomes (back arrows) and mitochondria in the myocardium of WT and DJ-1KO sham and 48 h post-CLP. Scale bar = 500 nm. Bars are mean + SEM of % mitochondria (per unit area cytoplasm). (**D**) Representative Western blot showing expression of p62, LC3-I, LC3-II and Rubicon protein in myocardium of WT and DJ-1KO mice at 24 and 48 h post-CLP or sham operation. Densitometry units are shown. *n* = 3/group. * *p* < 0.05, ** *p* < 0.01.

Sepsis-induced myocardial depression is associated with metabolic reprogramming, regulated by peroxisome proliferator-activated receptor gamma coactivator 1α (PGC-1α) [8]. PGC-1α mRNA and protein was decreased in WT and DJ-1 mice 24 h post-CLP (Figure 7A, B). In contrast, at 48 h post-CLP, PGC-1α mRNA and protein increased in both WT and DJ-1KO mice, although to a greater extent in DJ-1KO (Figure 7A,B). These findings collectively reveal that DJ-1KO mice had increased PGC-1α expression supporting a potential role for autophagy in DJ-1KO mice. Next, we explored whether autophagy pathways were involved in the improved pathogen clearance seen in DJ-1 deficient mice [27]. Autophagy has been shown to be upregulated with various stressors, including oxidative stress and inflammation. Upregulation of autophagy has been shown to be protective in experimental models of sepsis. Representative images from 48 h post-CLP showed round or oval double-stranded autophagosome structures (Figure 7C). The WT showed mitochondrial membrane swelling, and a loss of structural integrity and a decrease in mitochondrial content (per cent mitochondria) whereas the DJ-1K, demonstrated an increase in autophagic structures, mitochondrial swelling, minimal loss of mitochondrial integrity, and a similar mitochondrial content to the sham groups (Figure 7C). p62, a marker of autophagy flux, was lower in DJ-1KO hearts, suggesting proper completion of the autophagy cycle (Figure 7D). LC3 lipidation (LC3-II), a critical marker of autophagosome biogenesis, was increased in DJ-1KO hearts compared with WT hearts at 48 h after CLP surgery (Figure 7D). Rubicon is a Beclin 1 binding protein modulating autophagosome maturation and endocytic trafficking [28]. In bone marrow macrophages, we showed that DJ-1 deficiency induces a Rubicon-dependent increase in L3C-associated phagocytosis [29]. We assessed the expression of Rubicon in WT and DJ-1KO hearts following 48 h of CLP. Rubicon protein levels only decreased in the myocardium of WT mice at 48 h post-CLP (Figure 7D).

### 3.9. DJ-1 Deficient Cardiomyocytes Have an Attenuated Hypertrophic Phenotype and Less Apoptosis despite a Marked Inflammation in Response to LPS

**Figure 8:**
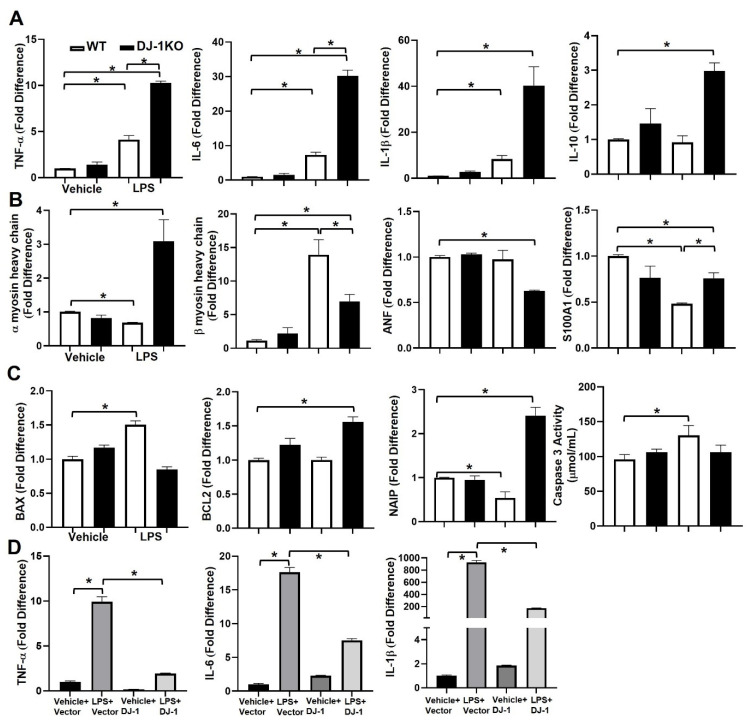
Loss of DJ-1 in cardiomyocytes exacerbates inflammation and represses hypertrophic signaling and apoptosis in response to LPS. (**A**) Bars are mean + SEM of TNF-α, IL-6, IL-1β, or IL-10 mRNA expression/18S in cardiomyocytes derived from the myocardium of WT and DJ-1KO mice at treated for 24 h with LPS (1 μg/mL). (**B**) Bars are mean + SEM of aMHC, bMHC, ANF, or S100A1 mRNA expression/18S in cardiomyocytes derived from the myocardium of WT and DJ-1KO mice treated for 24 h with LPS (1 μg/mL). (**C**) Bars are mean + SEM of BAX, BCL2, and NAIP mRNA expression/18S or caspase 3 activity (mmol/mL) in cardiomyocytes derived from the myocardium of WT and DJ-1KO mice treated for 24 h with LPS (1 μg/mL). (**D**) WT cardiomyocytes were transfected with a DJ-1 expression plasmid or empty vector and treated 24 h later with vehicle or LPS (1 μg/mL) for an additional 24 h. Bars are mean + SEM of TNF-α, IL-6, and IL-1β mRNA expression/18S. For all, *n* = 3/group. * *p* < 0.05.

The above results suggest that DJ-1 plays a regulatory role in CLP-induced myocardial depression in vivo. However, in addition to cardiomyocytes, DJ-1 is expressed in several cell types in the myocardium (i.e., smooth muscle) thus, the global loss-of-function strategy used above cannot rule out the contribution of nonmyocyte-DJ-1 in the regulation of myocardial dysfunction post-CLP. To specifically define the function of DJ-1 in cardiomyocytes, we cultured neonatal cardiomyocytes from WT and DJ-1KO hearts. Isolated cardiomyocytes were treated with the endotoxin LPS. LPS increased the mRNA expression of the inflammatory cytokine’s TNF-α, IL-6, and IL-1β in WT and DJ-1KO derived cardiomyocytes although to a greater extent in the DJ-1KO cultures (Figure 8A). The mRNA expression of the anti-inflammatory cytokine IL-10 was increased only in DJ-1KO derived cardiomyocytes in response to LPS (Figure 8A). Next, we examined the mRNA expression of the hypertrophic phenotypic genes αMHC, βMHC, and ANF. In response to LPS, the fetal βMHC was induced in WT cardiomyocytes whereas, the adult αMHC was markedly increased in DJ-1KO cardiomyocytes (Figure 8B). S100A1, the S100 protein associated with cardiomyocyte contractility, was markedly downregulated in WT compared to DJ-1KO cardiomyocytes in response to LPS (Figure 8B). LPS induced the upregulation of pro-apoptotic BAX in WT and upregulation of the anti-apoptotic markers BCL2 and XIAP in DJ-1deficient cardiomyocytes (Figure 8C). Additionally, LPS increased caspase 3 activity only in WT cardiomyocytes (Figure 8C). Next, we asked whether DJ-1 was regulating the inflammatory response. To evaluate this possibility, we overexpressed DJ-1 in cardiomyocytes derived from WT myocardium. As expected, LPS induced an increase in the expression of TNF-α, IL-6, and IL-1β, whereas overexpression of DJ-1 attenuated the LPS-induced inflammatory response (Figure 8D) Collectively, these results suggest that the loss of DJ-1 protects cardiomyocytes from LPS-induced apoptosis and hypertrophic fetal gene re-expression despite an increase in inflammation.

## 4. Discussions

We have shown mesenchymal stem cell (MSC) administration reduces sepsis-associated inflammation and multiple organ injury [37]. Microarray analysis of hearts from mice exposed to CLP and treated with MSCs demonstrated a reprogramming of the septic gene signature with an overall up regulation of mitochondrial related genes, and a downregulation of inflammatory markers and genes associated with Parkinson’s Disease. DJ-1 was one of the top downregulated genes in the myocardium after MSC administration. The evidence presented here supports a cardioprotective role of DJ-1 deficiency in sepsis-induced myocardial depression, despite an increased proinflammatory and pro-oxidant state in the myocardium. Firstly, archival myocardial biopsies from patients with sepsis-induced myocardial dysfunction have increased DJ-1 in the myocardium. Secondly, WT mice post-CLP also have enhanced myocardial DJ-1. Thirdly, DJ-1 deficient mice are resistant to sepsis-induced myocardial depression as assessed by increases in EF, FS, +dp/dt max, less fibrosis, decreased apoptosis, attenuated hypertrophic phenotype, and reduced mortality compared to WT mice. Loss of DJ-1 resulted in an increased ROS production enhancing bacterial phagocytosis and killing. Further, DJ-1 deficiency altered the balance between autophagy and phagocytosis (see below).

Septic myocardial depression, or septic cardiomyopathy, defined as global (systolic and diastolic) dysfunction in the heart, can be reversible [5,6,7,8]. It is a common finding in septic patients and is similarly observed in experimental models of sepsis [5,6,7,8]. The pathogenesis of sepsis-induced myocardial depression includes interaction of a combination of factors that result in autonomic and hemodynamic, molecular, metabolic, and structural alterations in the heart [6,38,39,40] An increase in ROS and inflammatory cytokines highlight myocardial depression. Intriguingly, in our study, DJ-1 deficient animals are resistant to sepsis-induced myocardial depression despite increased oxidative stress and inflammation. These findings and the consistency of our in vitro and in vivo data establish DJ-1 as a negative regulator of ROS production.

Sepsis is associated with downregulation of oxidative metabolism and cellular bioenergetics, which is associated with transcriptional switch in contractile and energy-related proteins [8]. The cell’s redox state plays a critical role in not just the regulation of oxidative stress and antioxidant defenses but cellular stress responses in general. The cellular redox state is coupled to mitochondrial biogenesis and repair network [41,42,43]. Activation of mitochondrial biogenesis via Nrf2-dependent and independent induction of heme oxygenase-1 have been shown to be critical in survival in sepsis [42]. In a S. aureus peritonitis model, induction of heme oxygenase-1 following carbon monoxide treatment activated mitochondrial biogenesis in the liver and improved survival [42]. Given that a decrease in/or loss of function of DJ-1 can result in an increased NRF2 degradation [44], NRF2 is not able to promote its antioxidant response, it was unexpected that DJ-1 deficient animals in our study showed upregulation of Nrf2 and Nrf2 dependent genes at 48 h, suggesting compensatory or non-canonical pathways of Nrf2 activation in our model. Moreover, DJ-1 deficient mice had increased recovery of PGC-1α expression. It is possible that Nrf2 is driving the protection, and DJ-1 does not contribute to the injury. Rather, the lack of DJ-1 creates an environment that increases oxidative stress to a level that drives Nrf2. Interestingly, overexpression of DJ-1 in cardiomyocytes attenuates the LPS-induced inflammatory response.

p62, an autophagy adaptor protein, upregulates Nrf2 expression and activity [45,46,47]. Phosphorylated p62 binds to Keap1, releasing Nrf2 to locate to the nucleus and induce transcription of cytoprotective genes [47]. The phosphorylated p62 along with Keap1 are subsequently targeted for autophagic degradation [48]. It has also been demonstrated that complete induction of autophagy in the hearts is protective in a CLP model of sepsis [49]. It is tempting to speculate DJ-1KO deficient mice may have increased Nrf2 activation via upregulation of autophagy pathways. Autophagy is induced by various cellular stressors, such as nutrient starvation, oxidative stress, ER stress, and inflammation. Induction of autophagy serves to preserve host homeostasis. Both decreased and increased autophagy have been demonstrated in experimental models of sepsis [49,50,51]. Suppressed autophagy is associated with increased mitochondrial dysfunction and tissue injury. Enhanced autophagy limits inflammation and promotes tissue repair. In a CLP model of sepsis, treatment of mice with rapamycin, an autophagy inducer, attenuated reduction in LVEF [49]. Similarly, administration of rapamycin and activated protein C in CLP model of sepsis limited lung inflammation and apoptosis, and subsequently improved survival [51]. Conversely, inhibition of autophagy in experimental sepsis models using rapamycin, chloroquine, 3-methyladenine, or VPS34 siRNA administration aggravated tissue injury and cell death (increased liver AST, hepatocyte apoptosis, reduced EF) [51,52,53]. Primary hepatocytes with knockdown of the autophagy associated gene Atg7 had augmented loss of albumin production and cell death [54]. Furthermore, p62 itself is regulated by Nrf2, thereby creating a positive feedback loop. Heme oxygenase-1 can also induce autophagy in the liver following CLP-induced sepsis. Inhibition of heme oxygenase, either pharmacologically using protoporphyrin or knockdown of heme oxygenase-1 prevented the induction of autophagy and enhanced hepatocellular injury and death [54]. Our findings show induction of autophagy as assessed by increased autophagosome formation, p62, LC3-I and -II, and the preservation of Rubicon in the myocardium of DJ-1KO mice compared to WT post-CLP surgery. This induction of autophagy may promote organ recovery and function.

Mechanistically, as in bone marrow macrophages, we propose that myocardial DJ-1 binding to p47^phox^ disrupts NADPH oxidase leading to Nox2 degradation thereby decreasing ROS production, impairing bacterial phagocytosis and killing [27]. Further, absence of DJ-1 may alter the balance between autophagy and phagocytosis [26]. In this regard, in DJ-1 deficient mice, the preservation of Rubicon drives non-canonical LC3-associated phagocytosis, facilitating bacterial killing in response to pathogen [29], resulting in the preservation of myocardial function. In contrast, in response to pathogen, in WT mice, increased DJ-1 degrades Rubicon driving canonical autophagy. We speculate that the ‘enhanced bacterial killing phenotype’ conferred by DJ-1 deficiency may in part explain the preservation of myocardial function and survival advantage. In this regard, Rubicon-deficient mice subjected to pressure overload by means of transverse aortic constriction developed heart failure with left ventricular dilatation, systolic dysfunction, and lung congestion [55]. Moreover, kidney-specific Rubicon KO mice developed metabolic syndrome suggesting a protective effect of Rubicon [56].

In conclusion, this study showed DJ-1 deficient mice are protected against CLP-induced myocardial depression despite increased oxidative stress and inflammation. Improved survival was accompanied by increased recovery of PGC-1α expression and non-canonical activation of Nrf2 antioxidant and p62-Rubicon-LC3 autophagy pathways.

## Data Availability

All data is available upon request.

## Abbreviations

CLP—cecal ligation and puncture; MSCs—mesenchymal stem cells; PD—Parkinson disease; MHC myosin heavy chain; Keap-1—Kelch Like ECH Associated Protein 1; Gpx1—Glutathione Peroxidase 1; GST—Glutathione s transferase; ANF—atrial natriuretic factor; IL—interleukin; NOS—nitric oxide synthase; LV—left ventricular; FS—fractional shortening; EF—ejection fraction; Hmox-1—heme oxygenase 1; LDH—lactate dehydrogenase; IL—interleukin; KC—keratinocyte chemoattractant; LPS—lipopolysaccharide; Nqo1—NAD(P)H Quinone Dehydrogenase 1; Nrf2—Nuclear Factor, Erythroid 2 Like 2; PARK7 (DJ-1)—Parkinson Disease (Autosomal Recessive, Early Onset) 7; NADPH—nicotinamide adenine dinucleotide phosphate; NLRP3—NOD-, LRR- and pyrin domain-containing protein 3; NOX2—NADPH oxidase 2; ROS—reactive oxygen species; TUNEL—terminal deoxynucleotidyl transferase dUTP nick end labeling; TNF—tumor necrosis factor; WT—wild type.
